# GSK3 Inhibitor-BIO Regulates Proliferation of Immortalized Pancreatic Mesenchymal Stem Cells (iPMSCs)

**DOI:** 10.1371/journal.pone.0031502

**Published:** 2012-02-23

**Authors:** Hui Cao, Yuankui Chu, Xiao Lv, Pubin Qiu, Chao Liu, Huiru Zhang, Dan Li, Sha Peng, Zhongying Dou, Jinlian Hua

**Affiliations:** 1 College of Veterinary Medicine, Shaanxi Center of Stem Cells Engineering and Technology, Key Lab for Animal Biotechnology of Agriculture Ministry of China, Northwest A & F University, Yangling, Shaanxi, People's Republic of China; 2 Hope Bio-technology (Su Zhou) Co., Ltd., Su Zhou, Jiangsu, People's Republic of China; 3 College of Bioengineering, Henan University of Technology, Zhengzhou Henan, People's Republic of China; George Mason University, United States of America

## Abstract

**Background:**

The small molecule 6-bromoindirubin-30-oxime (BIO), a glycogen synthase kinase 3 (GSK3) inhibitor, is a pharmacological agent known to maintain self-renewal in human and mouse embryonic stem cells (ESCs). However, the precise role of GSK3 in immortalized pancreatic mesenchymal stem cells (iPMSCs) growth and survival is not completely understood at present.

**Results:**

To determine whether this molecule is involved in controlling the proliferation of iPMSCs, we examined the effect of BIO on iPMSCs. We found that the inactivation of GSK3 by BIO can robustly stimulate iPMSCs proliferation and mass formation as shown by QRT-PCR, western blotting, 5-Bromo-2-deoxyuridine (BrdU) immunostaining assay and tunel assay. However, we did not find the related roles of BIO on β cell differentiation by immunostaining, QRT-PCR assay, glucose-stimulated insulin release and C-peptide content analysis.

**Conclusions:**

These results suggest that BIO plays a key role in the regulation of cell mass proliferation and maintenance of the undifferentiated state of iPMSCs.

## Introduction

Diabetes mellitus has now becoming one of the highest among chronic metabolic diseases which are heavily threatening people's health and can develop major damages to many systems and organs [1]. These syndromes put heavy burden on patients. Relative or absolute deficiency of pancreatic β-cell mass resulted in type I and type II diabetes occurrence [2]. Type I diabetes is a common endocrine disorder by a marked reduction in the number of pancreatic β-cells, resulting in substantial morbidity and mortality. Although daily insulin injections remains the most effective treatment for insufficient insulin secretion and abnormally high blood glucose levels from diabetes, it does not fully provide sufficient control of blood glucose that is exerted by endogenous β-cells [1], which has provided the impetus for intensive research to discover better methods of sustaining normoglycaemia. Previous reports have shown that transplantation of β-cells is an efficient approach to restore the insulin-secreting system and the precisely tune the insulin release in response to multiple neural and humoral signals arising within and beyond the islets of Langerhans [3]. However, the discrepancy between the limited number of donor islets and the high number of patients who could benefit from such a treatment reflects the need for renewable sources of high quality islet β-cells through other new methods [4]. The usage of porcine islet cells is currently viewed as one of the most promising alternatives not only due to the plenty supply of porcine islet cells, but also because porcine and human insulin are highly conserved and physiological glucose levels in porcine are similar to those in human [5]. The rationale for xenotransplantation is that the implanted porcine islets have the potential to mimic the normal physiological insulin response in type 1 diabetics, so that near-normal blood glucose levels are achievable without insulin administration or with a reduced requirement for it [6]–[7]. New islets can also be derived from pancreatic stem cells (PSCs). However, PSCs are rare and have a finite proliferative lifespan, culminating in permanent growth arrest, known as replicative senescence, resulting in the inability to multiply and phenotypic instability [8]. Immortalized pancreatic mesenchymal stem cells (iPMSCs) have been established and demonstrated these cells shared characteristics of typical bone marrow derived MSCs, ESCs, PSCs and unlimited potential of growth, possessed multipotent differentiation capacity and could differentiate into other functional cell types including neural, cardiomyocytes, even follicle like and islet-like cells by a specific method, which demonstrated that these cells may provide resources for regenerative medicine, tissue engineering and basic research [7]. Previous studies have found that some small molecules regulate the self-renewal of stem cells [9]–[11], which bring new approaches in studying the mechanisms of stem cells and promote their usage.

Glycogen synthase kinase 3 (GSK3), a serine/threonine kinase with two highly homologous isoforms, GSK3α and GSK3β, is a key regulator of numerous signaling pathways, such as Wnt/β-catenin, PI3K/Akt and Hedgehog (Hh) [12]. Upon activation of the canonical Wnt pathway, inhibition of GSK3 results in dephosphorylation of β-catenin leading to its nuclear accumulation. Studies showed that BIO is the first pharmacological agent, which is an inhibitor of GSK3, shown to maintain self-renewal in human and mouse ESCs [12]–[13]. BIO activates Wnt signaling and is known to sustain pluripotency of both human and mouse ESCs by inhibiting GSK3β [9]–[10]. Whether BIO can regulate the proliferation and differentiation of iPMSCs is still an issue [14]–[15].

In this study, we investigated the effects of BIO on iPMSC and found that the inactivation of GSK3 can robustly stimulate iPMSCs proliferation and mass formation, as shown by QRT-PCR, western blotting, 5-Bromo-2-deoxyuridine (BrdU) immunostaining assay and tunel assay. However, we did not find the related roles of BIO on pancreatic β cell differentiation derived from iPMSCs. These results suggested that GSK3 inhibitor-BIO plays a key role in the regulation of iPMSCs, cell mass proliferation, and maintenance of the undifferentiated state.

## Materials and Methods

### Culture of iPMSCs

iPMSCs were obtained from our laboratory [7]. Cells were subcultured with 0.25% (w/v) trypsin (Invitrogen, Carlsbad, CA, USA) when reaching 70–80% confluency. The culture medium (Low glucose-DMEM, Invitrogen), containing 15% FBS, 0.1 mM β-mercaptoethanol (Sigma), 2 mM glutamine (Invitrogen) and 100 mg/mL penicillin/streptomycin, was refreshed every 2–3 days [6]. BIO (Merck, Padge Road, Beeston Nottingham, NG9 2JR, UK) was added in culture medium to evaluate its effects on iPMSCs proliferation. Additionally, 25 ng/mL Wnt3a (R&D Systems, Inc. 614 McKinley Place NE Minneapolis, MN 55413), 50 ng/mL Dkk1(R&D Systems) alone or in combination with 1 µM BIO, and LiCl (1, 5, 10, 25 and 50 µM, R&D Systems) were added in medium to evaluate its effects on the proliferation of iPMSCs [16].

### In vitro proliferation assay of iPMSCs

iPMSCs were cultured in *vitro* in the presence (0.1, 0.5, 1.0, 1.5, 2.0 µM) or absence of BIO (Control, the same volume of DMSO as BIO group) in medium for 3 and 7 days and the number of cells were counted. The effects of BIO were based on the morphology and the number of colonies stained with Giemsa. The experiments were repeated for at least 3 times.

### Growth curve

iPMSCs in the presence (1.0 µM) or absence of BIO were seeded to 24-well plates at a density of 2×10^4^ cells/well. The proliferation ability was assessed by growth curve at an interval of 24 h. The cells were trypsinized and the cell number was determined for seven consecutive days (n = 3). Cell population doubling time (PDT) was calculated from the formula: PDT = [log_2_/(logN_t_−logN_0_)]×t, where N_t_ = the number of cells after t hours of culturing and N_0_ = number of cells seeded.

### Immunofluorescence

In the absence or presence of BIO (1.0 µM, 3 d), cells were fixed with 4% paraformaldehyde (PFA) for 10 min at room temperature. Cells were permeabilized with 0.1% Triton X-100 for 10 min, blocked with 10% goat serum in PBS at room temperature for 1 h, and incubated with the primary antibodies overnight at 4°C. The primary antibodies included PDX1 (1∶200, Abcam, Cambridge, MA,USA), Glut2 (1∶200, Millipore, Billenca, MA, CA, USA), C-Myc (1∶200, Chemicon, Temecula, CA, USA), PCNA (1∶200, Millipore), TERT (1∶200, Chemicon), β-catenin (1∶100, Santa Cruz, CA. U.S.A.), C-peptide (1∶200, Abcam), Insulin (1∶200, Chemicon) and E-cadherin (1∶100, Millipore). After three washes with PBS, the cells were incubated with the secondary antibodies at room temperature for 1 h, followed by three washes in the same buffer. They were then incubated with Hoechst33342(Sigma) or PI (Sigma) at room temperature for 5 min. Images were captured and analysed with a Leica fluorescent microscope. The percentage of PDX1, Glut2, C-Myc, PCNA, TERT and β-catenin positive staining in the absence or presence of BIO was made by manual counting under fluorescent microscope [17].

### RT-PCR

Total RNAs for RT-PCR analysis were extracted from iPMSCs cultured in the absence or presence of BIO (1.0 µM, 3 d), and induced iPMSCs using Trizol (Invitrogen). The cDNAs were synthesized based on 500 ng of RNA with a commercially available kit (TaKaRa, Biotech. Co. Ltd.). The PCR conditions included denaturation at 94°C for 5 min, followed by 30 repeated cycles of 95°C for 30 sec, 55°C–58°C for 30 sec and 72°C for 30–60 sec, and extension at 72°C for 10 min. The primers were designed based on the sequences of the open reading frame from the NCBI GenBank and synthesized by AuGCT Biotechnology (Beijing). The PCR primers and the length of the amplified products are shown in Table 1.

**Table 1:** The PCR primers and the length of the amplified products.

Primers	Forward	Reverse	Product Size(bp)	Tm
β-actin	5′-gcggcatccacgaaactac-3′	5′-tgatctccttctgcatcctgtc-3′	138	58°C
Pdx1	5′-gagcccgaggagaacaagc-3′	5′-tgacagccagctccaccc-3′	121	58°C
Ngn3	5′-gcgagttggcactgagca-3′	5′-aagctgtggtccgctatgc-3′	220	58°C
Mafa	5′-ttcagcaaggaggaggtcat-3′	5′-acaggtcccgctctttgg-3′	190	58°C
hTERT	5′-gtgtgctgcagctcccatttc-3′	5′-gctgcgtctgggctgtcc-3′	264	58°C
PCNA	5′-agtggagaacttggaaatggaa-3′	5′-gagacagtggagtggcttttgt-3′	154	58°C
C-Myc	5′-ctggtgggcgagatcatca-3′	5′-cactgccatgaatgatgttcc-3′	304	54°C
CyclinA	5′-tggctgtgaactacattga-3′	5′-acaaactctgctacttctgg-3′	136	50°C
CyclinD1	5′-tgaactacctggaccgct-3′	5′-caggttccacttgagyttgt-3′	212	50°C
CDK2	5′-gccaggagttacttctatgc-3′	5′-tggaagaaagggtgagcc-3′	180	53°C
Caspase3	5′-gaagaccatagcaaaaggag-3′	5′-tgtctcaataccacagtcca-3′	207	50°C
Glut2	5′-ttgccttggatgagttatgtga-3′	5′-gcgtggtccttgactgaaaa-3′	120 bp	58°C
Insulin	5′-aagcgtggcatcgtggag-3′	5′-tcaggactttattgggttttgg-3′	128	58°C

The PCR products were analysed in 2% agarose (Invitrogen) gel electrophoresis, stained with ethidium bromide (Invitrogen), and visualized under UV illumination.

### QRT-PCR

The QRT-PCR reactions were set up in 25 µL reaction mixtures containing 12.5 µL 1× SYBR@ PremixExTaqTM (TaKaRa, Biotech. Co. Ltd.), 0.5 µL sense primer, 0.5 µL antisense primer, 11 µL distilled water, and 0.5 µL template. The reaction conditions were as follows: 95°C for 30 sec, followed by 40 cycles of 95°C for 5 sec, and 58°C for 20 sec. All expression levels were normalized to β-actin in each well. Expression was quantified as the ratio of the mRNA levels obtained from iPMSCs in the absence or presence of BIO (1.0 µM, 3 d).

### Western Blotting

Total cell extracts were prepared from iPMSCs in the absence or presence of BIO (1.0 µM, 3 d) and fetal porcine pancreas, and proteins were extracted in 1×SDS-PAGE sample loading buffer. Total cell proteins were resolved by SDS-PAGE, transferred to PVDF membrane, and probed with β-actin (1∶1000, Beyotime, Haimen, Jiangsu, China), PDX1 (1∶1000, Abcam), C-Myc (1∶1000, Chemicon), PCNA (1∶1000, Millipore), TERT (1∶1000, Chemicon), β-catenin (1∶100, Santa Cruz), E-cadherin (1∶1000, Millipore), P-β-catenin (S33/S37/T41, 1∶1000, Bioworld). Horse-radish peroxidase-conjugated anti-rabbit or anti-mouse IgG was used as a secondary antibody (1∶1000, Beyotime). The detection was performed using the BM-Chemiluminescence blotting substrate (Roche, Shanghai, China).

### BrdU incorporation assay

The proliferation of iPMSCs was assayed by BrdU incorporation, performed similarly to previous reports [18], but with some modifications. First, iPMSCs in the absence or presence of BIO (1.0 µM, 3 d) were treated with 30 µg/mL BrdU (Sigma, St Louis, MO, USA) for 6 h and then subjected to BrdU immunostaining. More specifically, cells were fixed in 4% PFA for 15 min at room temperature and washed three times, for 10 min each with PBS (pH 7.4) containing 0.1% Triton X-100. The cells were then washed for three times in PBS (pH 7.4) alone. Anti-BrdU (1∶100; Santa Cruz) dissolved in 0.1 M PBS (pH 7.4) containing 5% normal goat serum was added and the cells were incubated overnight at 4°C. Cells were washed in PBS (pH 7.4) three times, and then incubated with the secondary antibody (FITC, Millipore 1∶500) for 1 h at room temperature. Three more washes were carried out and cells were visualized under a Leica fluorescent microscope and analysed for BrdU uptake. The rate of BrdU positive in the absence or presence of BIO was made by manual counting under fluorescent microscope [17].

### Luciferase assays

FOP-Flash plasmids (contain mutated Tcf binding sites), as a negative control, and TOP-Flash (contain functional Tcf binding sites) were transiently transfected into iPMSCs in the absence or presence of BIO (1.0 µM, 3 d) using Lipofectamin-2000 (Invitrogen) according to the manufacturer's instructions. Transfection was performed in OPTI-MEM (Invitrogen). After 6 h, OPTI-MEM was replaced by medium with or without BIO. Luciferase activity was measured using Luciferase Assay System (Promega, Madison, WI 53711 USA) according to the manufacturer's instructions (a luminometer TD-20, Turner Design), TOP-Flash activity was normalized against FOP-Flash activity.

### Tunel assay

Cells cultured with and without BIO were fixed with 4% PFA for 30 min at room temperature, washed twice with PBS and were permeated with 0.1% Triton X-100 for 10 min. The cells were then washed with PBS incubated with tunel reaction mix (Beyotime) for 60 min in the dark, and were analysed with a Leica fluorescent microscope. The rate of tunel positive in the absence or presence of BIO was made by manual counting under fluorescent microscope [17].

### Generation of islet-like clusters derived from iPMSCs

We used a two-step protocol to obtain islet-like cells. First, iPMSCs were dissociated with 0.05% trypsin at 37°C for 5 min and cultured in RPMI 1640/B27 medium with or without BIO (1.0 µM) supplemented with 1% BSA, 100 mM nicotinamide (NIC, Sigma), 10 ng/mL exendin-4 (Sigma), 1 mM sodium pyruvate (Invitrogen), 2 mM glutamine (Invitrogen), 1 mM β-mercaptoethanol (Sigma), 100 mg/mL penicillin/streptomycin, 20 ng/mL EGF (Millipore) and 20 ng/mL bFGF (Millipore) for 7 days. Cells were then cultured for another 7 days in the same medium supplemented with 10^−6^ M RA (Sigma) but without EGF and bFGF. The cells were transferred into ultra-low attachment plates and cultured in the same medium for 2 weeks. The medium was changed every 3 days.

The clusters were also identified by immunofluorescent assay with PDX1, C-peptide and Insulin antibodies. PDX1, Ngn3, Mafa, Glut2, PC1/3 and Insulin were analysed by QRT-PCR at day 14 after induction. Uninduced iPMSCs were used as negative control.

### Glucose-stimulated insulin release and insulin content analysis

After 14 day induction, iPMSC-derived islet clusters were plated onto 6-well plate in triplicate, approximately 200 clusters/well, and cultured overnight to ensure the clusters attached to the plate [7]. Then, the clusters were washed three times with glucose-free RPMI 1640 medium and the clusters were further incubated with RPMI 1640 medium containing 25 mM and 5.6 mM glucose at 37°C for another 2 h [19]. In this experiment, the different stages of clusters subsequently were incubated with 5.6 and 25 mM glucose for 2 h to determine glucose-stimulated insulin content and C-peptide release. The RPMI 1640 induction medium was determined as the basal level of insulin content and C-peptide release. The supernatant of both low- and high-glucose-treated clusters, and the untreated iPMSCs treated with low and high concentrations of glucose were collected and stored at −70°C for the further analysis. The insulin secretion and content derived the clusters was performed by RIA [18].

### Statistical analysis

The effects of BIO on the proliferation and apoptosis of iPMSCs were evaluated based on fluorescence staining, QRT-PCR, BrdU staining and tunel staining. The Data are presented as mean±SEM and the standard errors of the mean (SEM) in this study were calculated for 3 replicates in each of the 3 independent experiments [17]. Statistical comparisons were assessed with analysis of Student's test. P<0.05 was considered statistically significant difference and P<0.01 was considered highly significant difference.

## Results

### The GSK-3 inhibitor BIO promotes self-renewal of iPMSCs

**Figure 1:**
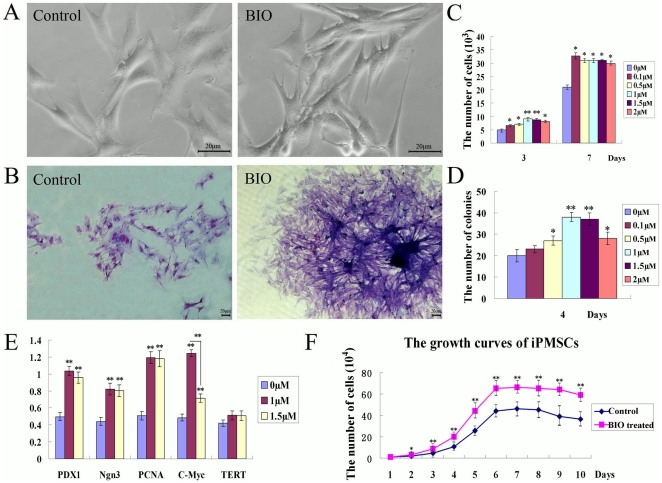
Morphology of iPMSCs in the absence or presence of BIO. A-Control, The typical spindle fibroblast-like morphology of iPMSCs; A-BIO, The morphology of iPMSCs treated with BIO; B, iPMSCs stained with Giemsa in the absence or presence of BIO; C, The number of cells cultured in the absence or presence of BIO (0.1, 0.5, 1.0, 1.5, 2.0 µM); D, The number of iPMSCs colonies; E, Expression of PDX1, PCNA, Ngn3, TERT and C-Myc were analysed at mRNA level in the absence and presence of 1.0 and 1.5 µM BIO; F, The growth curves of iPMSCs cultured in the absence and presence of BIO (1 µM). Bar = 20 µm. * P<0.05, **, P<0.01.

iPMSCs presented as the typical triangles and long spindle cell morphology of mesenchymal-like cells in the presence and absence of BIO (Figure 1A). Different concentrations of BIO (0, 0.1, 0.5, 1.0, 1.5, 2.0 µM) were added to the culture medium to evaluate their effects based on the morphology of iPMSCs colonies stained with Giemsa. Cell colonies were significantly denser and bigger in BIO than without BIO (Figure 1B). iPMSCs cultured in the presence of BIO (0.1, 0.5, 1.0, 1.5, 2.0 µM) for 3 and 7 days *in vitro* proliferated faster than that in the absence of BIO (Figure 1C). The number of cells in the presence of BIO (1.0, 1.5 µM) was elevated 1.2–2 fold compared with that in the absence of BIO. But when treated with 2.0 µM BIO, the number of iPMSCs decreased. In addition, the number of colonies was significantly increased (about 2 fold, P<0.01) in the presence of BIO (Figure 1D). The results showed that BIO could promote the proliferation of iPMSCs, and we concluded that the optimal concentrations were 1.0 and 1.5 µM. Then we detected specific pancreatic stem cell markers at the mRNA level. Expression of PDX1, Ngn3, PCNA and C-Myc showed an upward trend in iPMSCs treated with 1.0 and 1.5 µM BIO (Figure 1E) as compared with the control group. And the expression of C-Myc was clearly increased in the presence of 1.0 µM BIO compared with 1.5 µM and control. Thus, we used 1.0 µM BIO in the following experiments.

The growth curves of iPMSCs cultured in the absence and presence of BIO (1.0 µM) were shown in Figure 1F. The cells maintained in Lag phase for 1–2 days, for 3–6 days into the Log (exponential) phase (Figure 1F). The PDT of iPMSCs cultured in the absence and presence of BIO (1.0 µM) were 21.97 h *VS* 19.93 h (P<0.01) , respectively. These results showed that iPMSCs had a stronger proliferative capacity in the presence of BIO compared with that in the absence confirmed by growth curves (Figure 1F).

**Figure 2:**
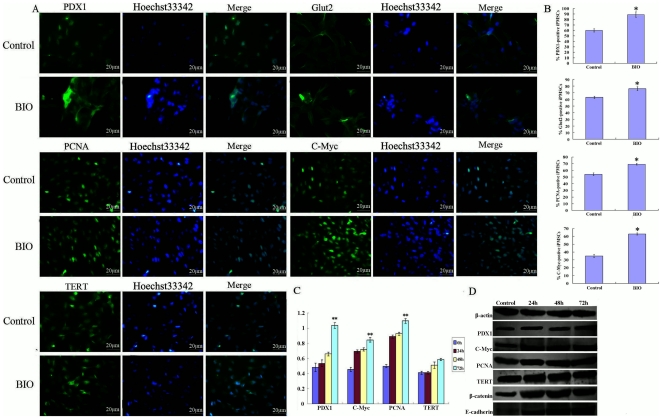
The GSK-3 inhibitor BIO promotes self-renewal of iPMSCs. A, Immunofluorescene staining analysis showed iPMSCs were positive for PDX1, Glut2, PCNA, C-Myc and TERT in the absence or presence of BIO, Bar = 20 µm; B, The percentage of positive cells treated with BIO or not from A; C, QRT-PCR analysis showed that the expression of pancreatic islets-specific marker PDX1 and pluripotent markers C-Myc, PCNA and TERT had an up-regulated trend in BIO cultured cells (24, 48, 72 h); D, Treated with BIO (24, 48, 72 h), western blotting analysis showed the expression of PDX1, PCNA, C-Myc, TERT and E-cadherin proteins had an upward trend compared with untreated cells. Bar = 20 µm, * P<0.05, **P<0.01.

The iPMSCs also expressed the characteristic surface markers of PSCs including PDX1 and Glut2 as well as the proliferative markers of TERT, C-Myc and PCNA (Figure 2A Control) as shown by immunofluorescence. When cells were seeded at a lower density, the iPMSCs in the presence of BIO were easier to form cell mass and expressed PDX1, Glut2, TERT, C-Myc and PCNA as shown by immunofluorescence staining (Figure 2A). The percentage of positive cells had an up-trend compared with that in the absence of BIO: PDX1, 89% *VS* 60%; Glut2, 80% *VS* 65%; PCNA, 70.8% *VS* 56.2%; C-Myc, 65% *VS* 37.5% (P<0.05, respectively), and the TERT-positive cells of iPMSCs in the presence of BIO were similar to that in the absence of BIO (P>0.05). The expression levels of the pancreatic stem cell specific marker PDX1, the proliferative markers C-Myc and PCNA were up-regulated in BIO medium compared with in the absence of BIO analysed by QRT-PCR (Figure 2C, P<0.01). Treated with BIO for 72 h, the expression of PDX1, C-Myc and PCNA were the highest, with the expression of PDX1 and C-Myc increasing about 2 fold. The expression of PDX1, PCNA, C-Myc and TERT protein also had an upward trend compared with untreated cells by western blotting (Figure 2D). We also found Wnt3a promoted the proliferation of iPMSCs (P<0.05) and increased the expression of PCNA, C-Myc and PDX1. However, Dkk1 inhibited the proliferation of iPMSCs (P<0.05) and the expression of C-Myc and PDX1 (Figure S1). The expression of cell cycle proteins: CyclinA and CyclinD were increased in BIO treated cells (Figure S2). Wnt3a and Dkk1 effect on the proliferation of iPMSCs were investigated, and we found Wnt3a play a role in mediating the effects of BIO and Dkk-1 inhibit the effects of BIO (P<0.05) (Figure S3). LiCl (5 and 10 µM) also stimulated the proliferation of iPMSCs, however, 25 and 50 µM LiCl inhibited their proliferation (P<0.05) and the expression of PDX1, C-Myc and PCNA (P<0.05) (Figure S4).

**Figure 3:**
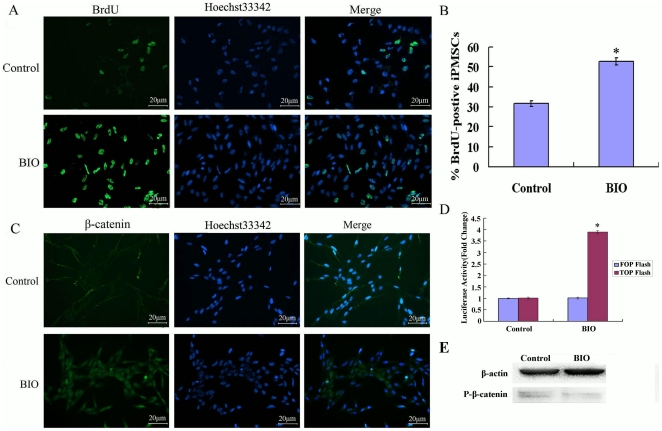
β-catenin and BrdU incorporation assay. A, The mitiosis index with BIO was significantly higher than without BIO medium; B, The percentage of positive cells treated with BIO or not from A; C, BIO treatment caused nuclear accumulation of β-catenin, which was not observed in control cells; D, Cells were assayed for β-catenin-mediated transcriptional activation by using a reporter containing either functional (TOP-Flash) or mutated (FOP-Flash) Tcf binding sites. Cells stimulated with BIO showed rise in the levels of β-catenin activity, as measured by luciferase activity. Treatment with BIO resulted in the higher fold change (almost 4 fold) when TOP-Flash activity was normalized against FOP-Flash activity. There was no significant difference in activities in the presence of the mutated FOP-Flash reporter; C, Bar = 20 µm, *P<0.05; E, Western blotting showed that p-β-catenin (S33/37/T41) was decreased in BIO treated.

BrdU incorporation assay demonstrated that the mitosis index in the presence of BIO was significantly higher than without BIO (Figure 3A, P<0.05). Control cells had a lower rate of BrdU incorporation (31.7±1.3%). In contrast, stimulation of iPMSCs with 1 µM BIO increased the BrdU incorporation rate to 52.7±1.9% (Figure 3B). These results demonstrated that BIO enhanced the proliferation of iPMSCs.

Meanwhile, β-catenin was activated when iPMSCs were cultured in the presence of BIO (Figure 3C). In control cells, β-catenin staining was weak and appeared to be located at the cell membrane. In contrast, BIO-stimulated cells showed nuclear staining, suggesting that there was an increased level of β-catenin (Figure 3C, Figure S5). Similarly, the expression of the β-catenin protein was increased in the presence of BIO, as shown by western blotting (Figure 2D). The β-catenin/Tcf activity driven from the TOP-Flash luciferase reporter containing functional Tcf-binding sites transfected into iPMSCs was enhanced after 3 days in 1.0 µM BIO treatment, and no activation occurred upon transfection with FOP-Flash reporter containing mutated Tcf-binding sites. Treatment with 1.0 µM BIO resulted in a higher fold change (almost 4 fold, P<0.01) when TOP-Flash activity was normalized against FOP-Flash activity (Figure 3D). There was no significant difference in activities in the presence of the FOP-Flash reporter. Western blotiing showed that the level of P-β-catenin (S33/S37/T41) was decreased in BIO treated (Figure 3E).

**Figure 4:**
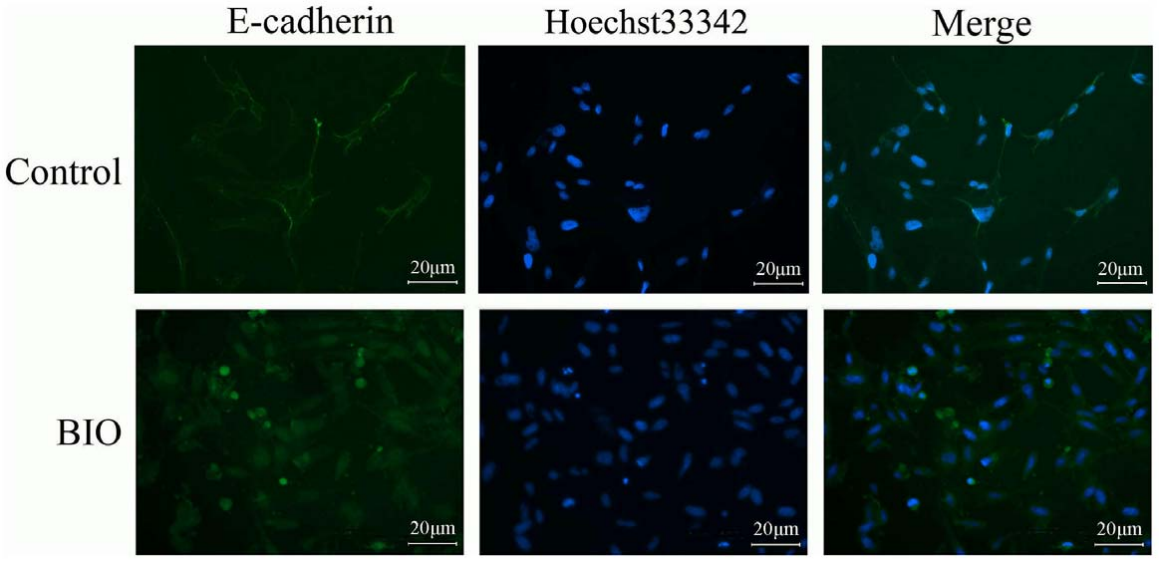
E-cadherin staining analysis. E-cadherin showed a bright membrane staining in untreated (Control) and translocated into cytoplasm in BIO-treated iPMSCs. Bar = 20 µm.

After treatment with 1.0 µM BIO, iPMSCs were more compact morphology and aggregated, suggesting a change in cell adhesiveness [20]. So we studied the intracellular localization and the level of E-cadherin upon BIO stimulation. As visualized by immunofluorescence, E-cadherin showed a bright membrane staining in untreated iPMSCs, and the expression of E-cadherin protein was present cytoplasm staining in BIO-treated IPMSCs (Figure 4).

**Figure 5:**
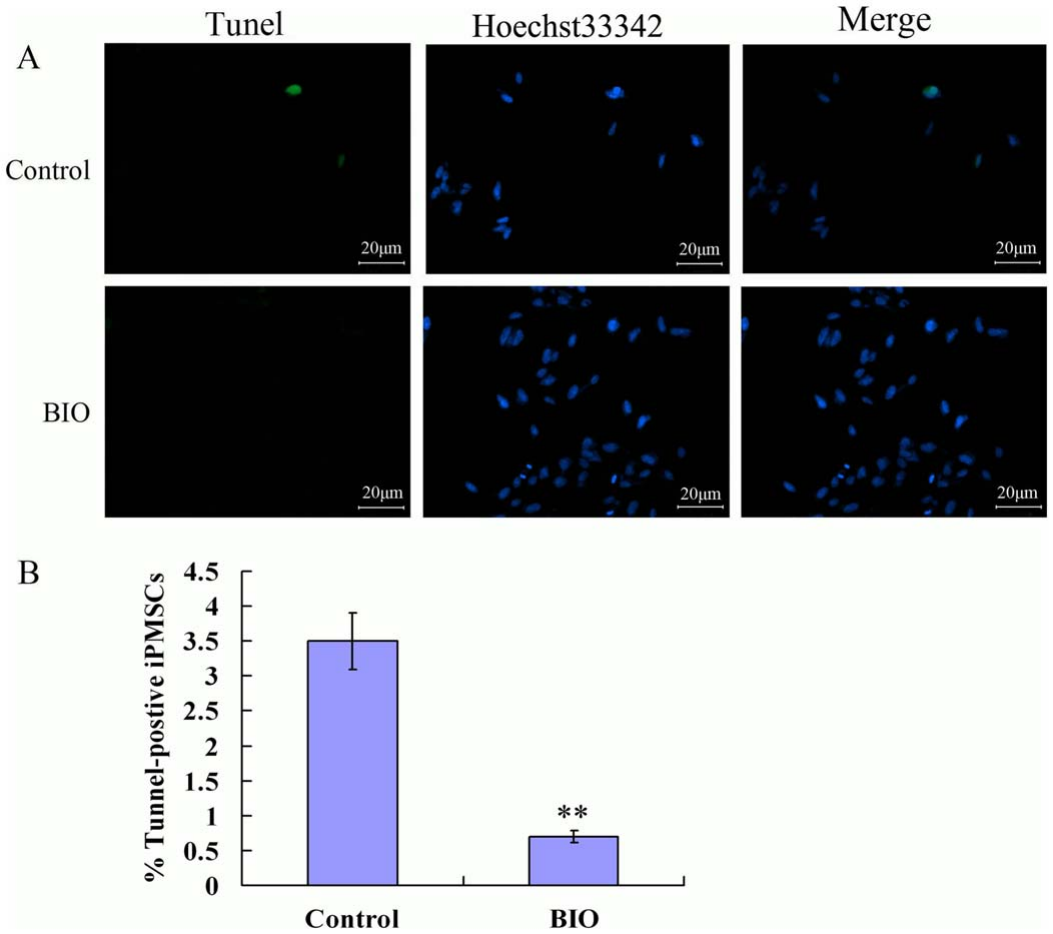
Tunel staining analysis iPMSCs cultured with BIO or not. Bar = 20 µm, **P<0.01.

Nearly no or little tunel positive cells were observed in BIO-supplemented medium. However, some tunel positive cells were observed (about 3.5%) in the absence of BIO (Figure 5, P<0.01).

### Differentiation potential of islets

**Figure 6:**
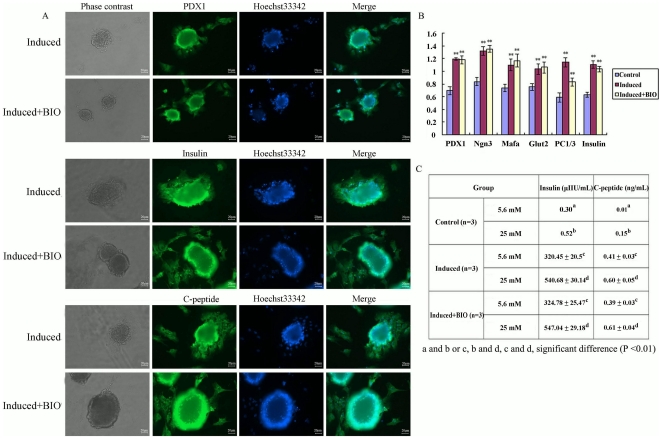
Potential differentiation of islet cells. A, The induced clusters treated with BIO or not were transferred into plates and stained positive for PDX1, C-peptide and insulin analysed by immunofluorescent staining; B, The specific markers of pancreatic islets including PDX1, Ngn3, Mafa, Glut2, PC1/3 and Insulin expressed in the iPMSCs treated with BIO or not by QRT-PCR; C, Glucose-stimulated insulin release and insulin content analysis. Bar = 20 µm, **P<0.01.

BIO stimulated iPMSCs to form smaller, denser and more compact colonies (Figure 1). Further, we investigated the islet potential differentiation of iPMSCs in the absence or presence of BIO by inducing the cells with a two-step protocol [7]. On the 14^th^ day, induced pancreatic islet-like clusters were formed. Islet-like formation derived from iPMSCs in containing BIO induced medium were denser (Figure 6). After two weeks, the induced clusters were transferred into plates and stained positive for PDX1, C-peptide and Insulin, as analysed by immunofluorescent staining (Figure 6A). These results provide evidences that the cells in the induction medium with or without BIO have the potential to differentiate into islet cell clusters and endocrine cell types in this protocol [21], and the number of islet cell clusters derived from iPMSCs were slightly increased in BIO induced medium. At the mRNA level, we detected endocrine pancreatic islet markers including PDX1, Ngn3, Mafa, Glut2, PC1/3 and Insulin expressed in iPMSCs treated with or without BIO, however, BIO treatment did not alter the expression levels of PDX1, Ngn3, Mafa, Glut2, PC1/3 and Insulin analysed by QRT-PCR (Figure 6B).

To determine whether the clusters derived iPMSCs in the presence or absence of BIO are capable of secretion insulin in response to glucose stimulation, we tested the ability of iPMSC-derived islet clusters for C-peptide production after sequential treatment with low and high concentrations of glucose in a static assay. The insulin content and C-peptide release levels in 25 mM and 5.6 mM glucose stimulated group for islets induced derived from iPMSCs in the presence and absence of BIO were significantly higher than that in control cells (P<0.01), and there were significantly different levels of the secreted insulin and C-peptide between 25 mM and 5.6 mM glucose stimulated respectively (P<0.05), however, there weren't significantly differences between in the presence and in absence of BIO (Figure 6C, P>0.05). This suggests that the GSK3 inhibitor- BIO can maintain the cells in an undifferentiated state, but not affect the potentiality of differentiation into pancreatic islet.

## Discussion

In this study, our results demonstrated that these iPMSCs proliferated better in the presence of BIO. We found that the pluripotent iPMSCs could be successfully cultured in BIO medium and BIO up-regulated the expression of proliferation markers including C-Myc, TERT and PCNA compared with control. Moreover, several cell cycle regulators, known to regulate the cell cycle progression during the G1/S transition, were also detected. Both Cyclin D1 and Cyclin A were up-regulated by BIO, which indicated that both of them were involved in regulating cell cycle progression from G1 to S phase induced by BIO. Simultaneously, these cells maintained an expression of PSC markers similar to the control group [7]. Myc is activated through various mitogenic signals such as Wnt, Shh and EGF (via the MAPK/ERK pathway). Myc activation results in numerous biological effects including driving cell proliferation (by up-regulating cyclins), regulating cell growth (by up-regulating ribosomal RNA and proteins), apoptosis, differentiation and self-renewal of stem cells. Myc is a very strong proto-oncogene and its over-expression stimulates gene amplification [22]. Proliferating cell nuclear antigen (PCNA) is a distinct marker for the proliferative cell [23]–[24], which was up-regulated in BIO medium. These results demonstrated that BIO may regulate C-Myc, which results in the up-regulation of PCNA, CDK2, CyclinA and CyclinD1 to maintain the self-renewal of iPMSCs. Our results have demonstrated that Wnt3a function in mediating the effects of BIO, while Dkk-1 inhibit the effects of BIO. However, LiCl has different effect on iPMSC proliferation at different concentration. GSK3β is the best-studied target of Li [25]. LiCl promotes the reprogramming process and the generation of induced pluripotent stem cells (iPSCs). The results showed that the greatest effect of LiCl was 10 mM. Prolonged treatment of LiCL resulted in the reduction in the colony size and eventual reduction in the number of colonies of iPSCs because of a cytotoxic effect [25]. Different concentration of Li affect the potential of differentiation and proliferation of stem cells [26]–[27]. Whether the true mechanism of BIO in iPMSCs is similar to that in pluripotent stem cells or not, we need to further study the PI3K/Akt and the MEK/ERK pathway, epigenetics and the reprogramming mechanisms. The expression of PDX1 and C-Myc protein was increased by GSK3 inhibitor-BIO analysed by western blotting, which was consistent with previous studies [28]–[30]. However, sometime contradictory effects of GSK3 via the phosphorylation of some proteins were complex. Rocques et al (2007) found that Maf-transforming activity is controlled by GSK-3-dependent phosphorylation and that phosphorylation by GSK-3 can increase the oncogenic activity of a protein [31].

Maintenance of self-renewal potential is currently a challenge for *in vitro* culture of PSCs because these tissue stem cells isolated from the *in vivo* niche rapidly undergo apoptosis in culture if they lose their suitable microenvironment. Some growth factors and small molecules are known to be essential for the self-renewal of pluripotent stem cells [15]. Wnt consists of a large family of secreted glycoproteins and extracellular Wnt-ligands, which interact with receptors of the frizzled family, involved in cell proliferation, differentiation, organogenesis, and cell migration [32]–[33]. The function of GSK3 inhibitor-BIO in the culture of pluripotent stem cells has been previously described [9]–[10]. Studies have also demonstrated that BIO stimulated the proliferation of β cells and cardiomyocytes in vitro [2], [17]. Engagement of Wnt ligands with the receptor activates the Dishelved (Dsh) protein. Dsh inhibits GSK-3 which leads to Wnt signaling phosphorylating and targeting the β-catenin-Adenomatous Polyposis Coli (APC) complex for ubiquitination and proteolytic degradation [12]. Upon Wnt signaling, β-catenin is stabilized, accumulates in the cytoplasm and is eventually translocated to the nucleus, where it interacts with DNA-binding proteins of the T-cell Factor/Lymphocyte Enhancer binding Factor (Tcf/Lef) family. In the presence of β-catenin, Tcf/Lef act as transcriptional activity [31]. However, BIO may inhibit GSK3β activity, and activate Wnt/β-catenin signaling and Ca^2+^ signaling pathways [34]–[35]. In this study, β-catenin and E-cadherin were translocated into the nucleus or cytoplasm, and the total β-catenin was increased in BIO treated. The accumulation of β-catenin is a clear hallmark of Wnt signaling [36]. Meanwhile, p-β-catenin (S33/37/T41) was decreased in BIO treated analysed by Western blotting. These results clearly demonstrated that BIO inhibited GSK3β activity, and activate Wnt/β-catenin signaling and Ca^2+^ signaling pathways in iPMSCs [20]. The key downstream transcriptional regulators of the Wnt pathway influence transcription of target genes including C-Myc, TERT and CDK2, thus, promoting the proliferation of iPMSCs. Our results first demonstrated that BIO enhances the proliferation of iPMSCs, activates β-catenin signaling, and increases the proliferative markers including TERT, C-Myc and PCNA, as shown by cell morphology, mRNA and protein levels while it suppresses apoptosis, as shown by tunel staining and BrdU assay. Meanwhile, the cultured iPMSCs in BIO maintained the characteristics of iPMSCs. These results were consistent with previous studies, which have shown that BIO promote the proliferation of iPMSCs through regulation of the target genes of β-catenin signaling in β cells and cardiomyocytes [2], [9], [15], [17], [28]–[30]. Furthermore, our research found that iPMSCs maintained the undifferentiated state in BIO medium, but did not change their potentiality to differentiate into insulin producing cells. Therefore, we report for the first time that BIO may stimulate iPMSCs self-renewal *in vitro*. However, we need to elucidate the molecular mechanisms.

## Supporting Information

Figure S1 **Wnt3a and Dkk1 effect on the proliferation of iPMSCs.** A, The number of cells treated by 25 ng/mL Wnt3a, 50 ng/mL Dkk1 alone (P<0.05); B, Expression of PDX1, C-Myc, PCNA and TERT were analysed at mRNA level treated by 25 ng/mL Wnt3a, 50 ng/mL Dkk1 alone; C, Expression of PDX1, C-Myc, PCNA and TERT were analysed at protein level treated by 25 ng/mL Wnt3a, 50 ng/mL Dkk1 alone.
(TIF)

Figure S2 **RT-PCR analysis the effects of BIO on the expression of cell cycle proteins in iPMSCs.**
(TIF)

Figure S3 **The number of iPMSCs treated by 25 ng/mL Wnt3a, 50 ng/mL Dkk1 in combination with 1 µM BIO (P<0.05).**
(TIF)

Figure S4 **The effect of LiCl on the proliferation of iPMSCs.** A, The number of iPMSCs treated with LiCl (1, 5, 10, 25 and 50 µM); B, Expression of PDX1, C-Myc and PCNA were analysed at mRNA level treated by LiCl (1 µM, 5 µM and 10 µM) (P<0.05).
(TIF)

Figure S5 **BIO treatment resulted in nuclear accumulation of β-catenin, which was not observed in control cells.** The nucleus was stained with PI.
(TIF)
